# Crystal structure of l-tryptophan–fumaric acid–water (1/1/1)

**DOI:** 10.1107/S205698901501484X

**Published:** 2015-08-15

**Authors:** M. Lydia Caroline, S. Kumaresan, P. G. Aravindan, M. Peer Mohamed, G. Mani

**Affiliations:** a PG & Research Department of Physics, Arignar Anna Govt. Arts College, Cheyyar 604 407, Tamil Nadu, India; b Thiru.Vi.Ka. Govt Arts College, Thiruvarur 610 003, Tamilnadu, India; c Department of Physics, C. Abdul Hakeem College, Melvisharam 632 509, Tamil Nadu, India

**Keywords:** crystal structure, l-tryptophan, fumaric acid, hydrogen bonding, three-dimensional structure

## Abstract

In the title compound, C_11_H_12_N_2_O_2_·C_4_H_4_O_4_·H_2_O, the l-tryp­to­phan mol­ecule crystallized as a zwitterion, together with a neutral fumaric acid mol­ecule and a water solvent mol­ecule. In the crystal, the three components are linked by a series of N—H⋯O, O—H⋯O and C—H⋯O hydrogen bonds, forming slabs lying parallel to (001). The slabs are connected by O—H⋯O hydrogen bonds, involving inversion-related fumaric acid groups, leading to the formation of a three-dimensional structure.

## Related literature   

For literature on the UV spectroscopy of proteins, see: Demchenko (1986 ▸). For the different polymorphic forms of fumaric acid, see: Reis & Schneider (1928 ▸); Yardley (1925 ▸); Bednowitz & Post (1966 ▸). For the nonlinear optical properties of organic mol­ecules, see: Chemla & Zyss (1987 ▸); Zyss & Ledoux (1994 ▸); Zyss & Nicoud (1996 ▸). For the common conformations of l-tryptophan, see: Bye *et al.* (1973 ▸); Bakke & Mostad (1980 ▸). The bond lengths and angles in l-trypophan, see, for example: Gorbitz (2006 ▸); Gorbitz *et al.* (2012 ▸), and for fumaric acid, see: Goswami *et al.* (1999 ▸). For the crystal structure of l-tryptophan formic acid solvate, see: Hubschle *et al.* (2002 ▸). For details of the Cambridge Structural Database, see: Groom & Allen (2014 ▸).
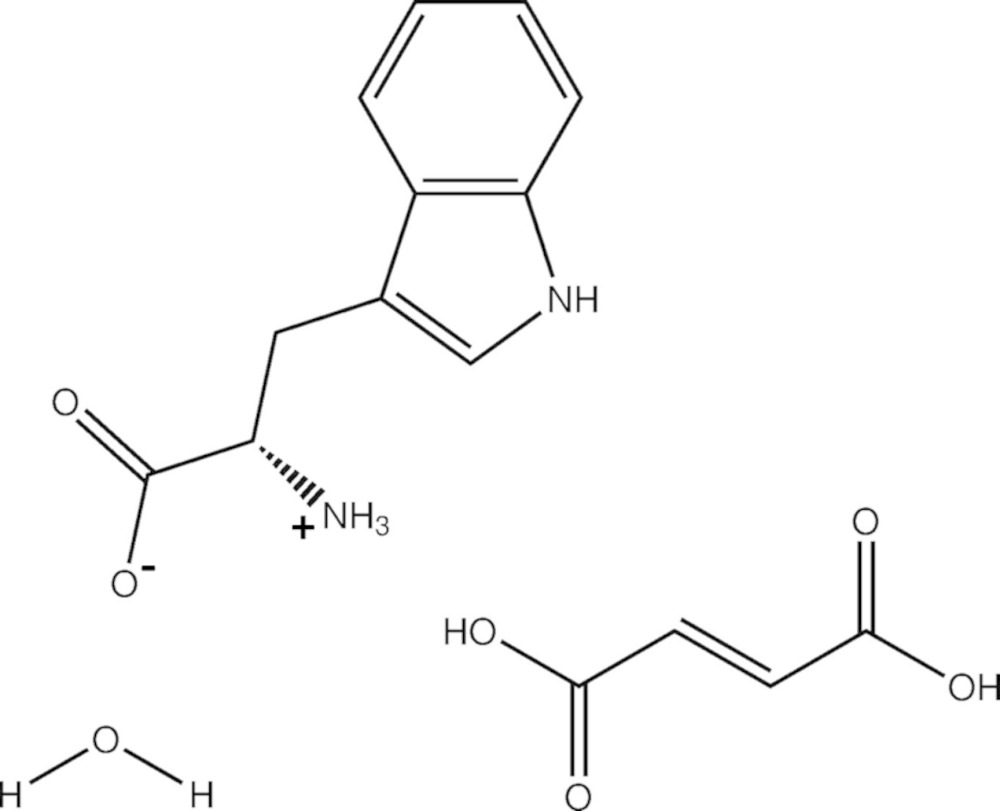


## Experimental   

### Crystal data  

C_11_H_12_N_2_O_2_·C_4_H_4_O_4_·H_2_O
*M* *_r_* = 338.31
Monoclinic, 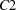

*a* = 11.3928 (8) Å
*b* = 6.6476 (4) Å
*c* = 21.4219 (13) Å
β = 95.801 (3)°
*V* = 1614.07 (18) Å^3^
*Z* = 4
Mo *K*α radiation
μ = 0.11 mm^−1^
*T* = 296 K
0.30 × 0.20 × 0.20 mm

### Data collection  

Bruker Kappa APEXII CCD diffractometer
Absorption correction: multi-scan (*SADABS*; Bruker, 2004 ▸) *T*_min_ = 0.898, *T*_max_ = 0.978
10737 measured reflections
3157 independent reflections
2731 reflections with *I* > 2σ(*I*)
*R*_int_ = 0.025

### Refinement  

*R*[*F* ^2^ > 2σ(*F* ^2^)] = 0.034
*wR*(*F* ^2^) = 0.084
*S* = 1.04
3157 reflections
242 parameters
4 restraints
H atoms treated by a mixture of independent and constrained refinement
Δρ_max_ = 0.18 e Å^−3^
Δρ_min_ = −0.15 e Å^−3^

### 

Data collection: *APEX2* (Bruker, 2004 ▸); cell refinement: *APEX2* and *SAINT* (Bruker, 2004 ▸); data reduction: *SAINT* and *XPREP* (Bruker, 2004 ▸); program(s) used to solve structure: *SIR92* (Altomare *et al.*, 1994 ▸); program(s) used to refine structure: *SHELXL2014* (Sheldrick, 2015 ▸); molecular graphics: *ORTEP-3 for Windows* (Farrugia, 2012 ▸) and *Mercury* (Macrae *et al.*, 2008 ▸); software used to prepare material for publication: *SHELXL2014* and *PLATON* (Spek, 2009 ▸).

## Supplementary Material

Crystal structure: contains datablock(s) I, global. DOI: 10.1107/S205698901501484X/su5176sup1.cif

Structure factors: contains datablock(s) I. DOI: 10.1107/S205698901501484X/su5176Isup2.hkl

Supporting information file. DOI: 10.1107/S205698901501484X/su5176Isup3.cml

**Fig. 1.**
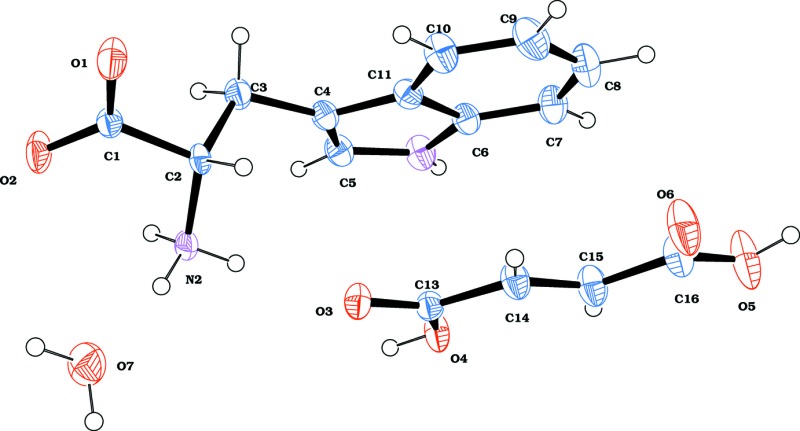
The molecular structure of the title compound, with atom labelling. Displacement ellipsoids are drawn at the 40% probability level.

**Fig. 2.**
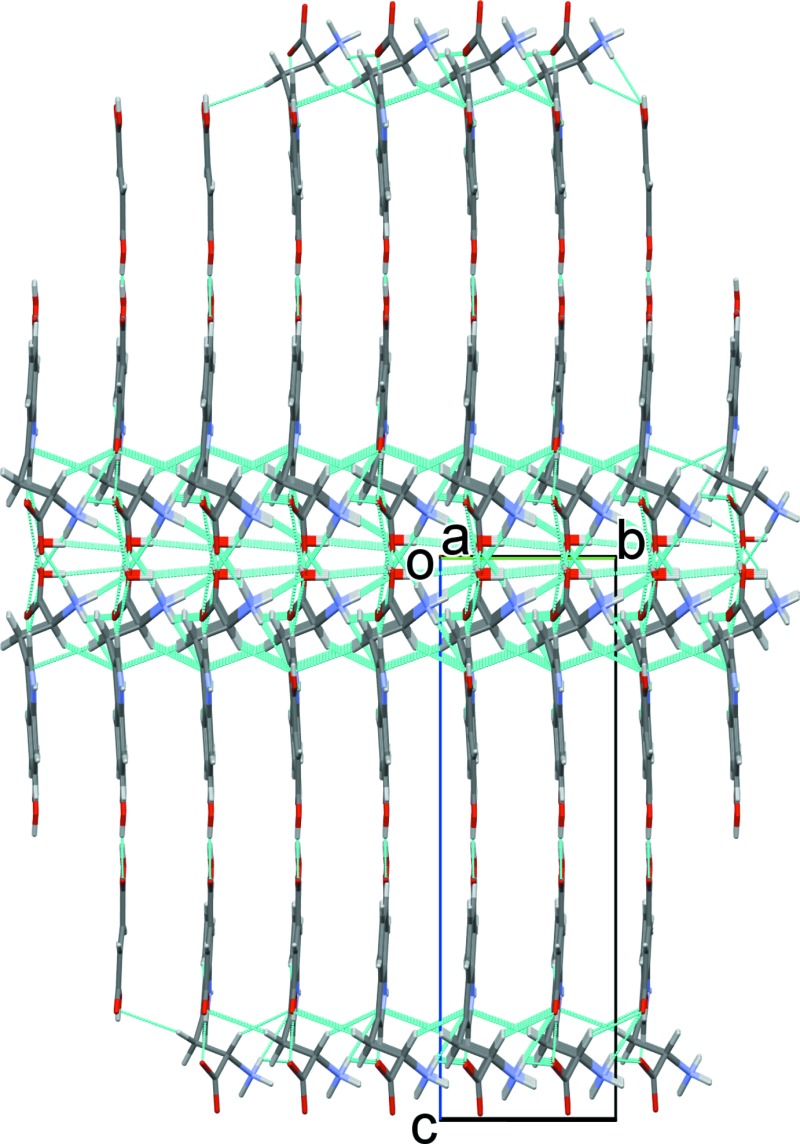
The crystal packing of the title compound, viewed along the a axis. The hydrogen bonds are shown as dashed lines (see Table 1 for details).

**Fig. 3.**
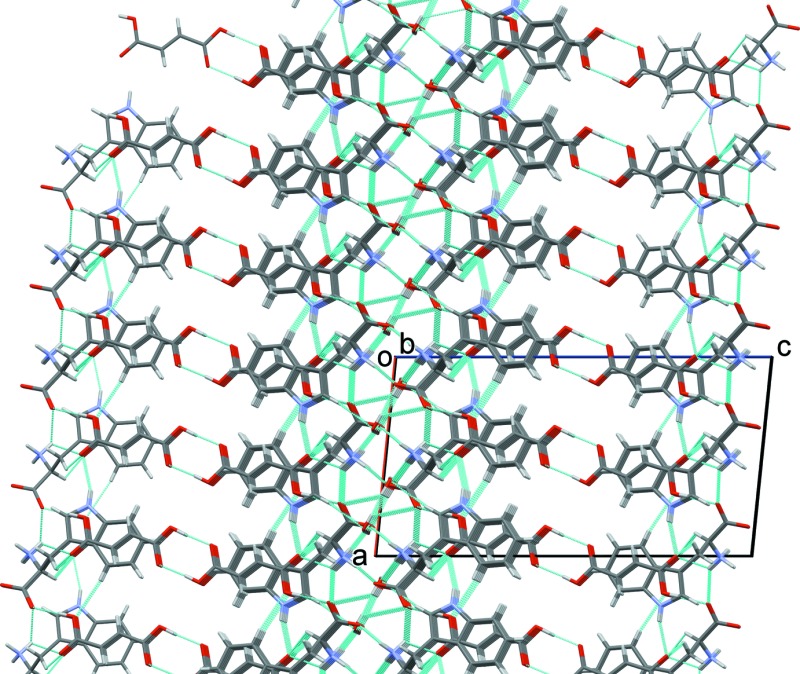
The crystal packing of the title compound, viewed along the b axis. The hydrogen bonds are shown as dashed lines (see Table 1 for details).

. DOI: 10.1107/S205698901501484X/su5176fig1.tif
The mol­ecular structure of the title compound, with atom labelling. Displacement ellipsoids are drawn at the 40% probability level.

a . DOI: 10.1107/S205698901501484X/su5176fig2.tif
The crystal packing of the title compound, viewed along the *a* axis. The hydrogen bonds are shown as dashed lines (see Table 1 for details).

b . DOI: 10.1107/S205698901501484X/su5176fig3.tif
The crystal packing of the title compound, viewed along the *b* axis. The hydrogen bonds are shown as dashed lines (see Table 1 for details).

CCDC reference: 1417535

Additional supporting information:  crystallographic information; 3D view; checkCIF report

## Figures and Tables

**Table 1:** Hydrogen-bond geometry (, )

*D*H*A*	*D*H	H*A*	*D* *A*	*D*H*A*
N1H1O3^i^	0.85(3)	2.11(3)	2.912(3)	158(3)
N2H2*A*O7	0.92(4)	1.94(4)	2.845(3)	170(3)
N2H2*B*O1^ii^	0.94(3)	2.30(3)	3.085(3)	140(2)
N2H2*B*O3	0.94(3)	2.28(3)	2.901(3)	123(2)
N2H2*C*O2^iii^	0.96(3)	1.87(3)	2.832(3)	174(3)
O4H4*O*O1^ii^	0.82	1.74	2.559(2)	178
O5H5*O*O6^iv^	0.82	1.81	2.630(3)	174
O7H7*A*O1^v^	0.88(2)	2.60(3)	3.261(3)	133(3)
O7H7*A*O2^v^	0.88(2)	1.97(2)	2.824(3)	165(3)
O7H7*B*O2^vi^	0.85(2)	2.53(3)	3.347(3)	162(3)
C3H3*B*O3^vii^	0.97	2.66	3.255(3)	120
C5H5O7^i^	0.93	2.58	3.491(3)	166

Symmetry codes: (i) 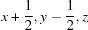; (ii) 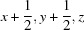; (iii) 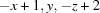; (iv) 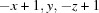; (v) 
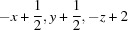
; (vi) 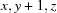; (vii) 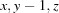.

## supplementary crystallographic information

### S1. Comment

Natural aromatic amino acids, particularly tryptophan, have near UV absorption and emission properties which are utilized extensively in solution phase investigations of structure-function relationships (Demchenko, 1986). Fumaric acid is known to exist in two different polymorphic forms viz. *cis* and *trans* ( Reis & Schneider, 1928; Yardley, 1925; Bednowitz & Post, 1966).

In conjunction with our ongoing work on non-linear optical organic crystals among the 20 naturally occurring amino acids, we have directed our inter­est to tryptophan (Trp), one of the essential amino acids for humans. The non-linear optical properties of organic molecules and crystals have been reviewed by Zyss (Chemla & Zyss, 1987; Zyss & Ledoux, 1994; Zyss & Nicoud, 1996). For similar properties and most the common confirmations of L-tryptophan have been reported (Bye *et al.*, 1973; Bakke & Mostad, 1980). Compared with other amino acids, there are less than 30 tryptophan structures listed in the Cambridge Structural Database (Groom & Allen, 2014), due to the difficulty of obtaining good optical quality crystals; as noted by (Hubschle *et al.*, 2002) who studied the crystal structure of *L*-tryptophan formic acid solvate. We successfully obtained good quality hard golden-yellow single crystals of *L*-tryptophan fumaric acid monohydrate, and we report herein on its synthesis and crystal structure.

In the title compound, Fig. 1, *L*-tryptophan is zwitterionic, as are most amino acids in the solid state, and fumaric acid is neutral. The bond lengths and angles in *L*-trypophan and fumaric acid are similar to those reported previously (Gorbitz, 2006; Gorbitz *et al.*, 2012; Goswami *et al.*, 1999).

In the crystal, the three components are linked by a series of O—H···O, N—H···O and C—H···O hydrogen bonds forming slabs lying parallel to (001); Table 1 and Fig. 2. The slabs are connected by O—H···O hydrogen bonds, involving inversion related fumaric acid groups, leading to the formation of a three-dimensional structure; Table 1 and Fig. 3.

### S2. Synthesis and crystallization

An aqueous solution of *L*-tryptophan and fumaric acid in a 1:1 stoichiometric ratio was stirred at room temperature for 6 h. The resulting yellow solution was filtered and kept in a Petri dish. Yellow prismatic-shaped hard crystals suitable for X-ray analysis were obtained over a period of 5 days.

### S3. Refinement

Crystal data, data collection and structure refinement details are summarized in Table 2. The N-bound, acid and water H atoms were located in a difference Fourier map. The NH and NH_3_ H atoms were freely refined. The water H atoms were refined with distance restraints: O—H = 0.86 (2) Å, H···H = 1.388 (20) Å with U_iso_(H) = 1.5U_eq_(O). The acid (OH) H atoms and the C-bound H atoms were included in calculated positions and treated as riding atoms: U_iso_(H) = 1.5U_eq_(O,C) for OH and methyl H atoms and 1.2U_eq_(C) for other H atoms.

### Crystal data

**Table:**

C_11_H_12_N_2_O_2_·C_4_H_4_O_4_·H_2_O	*F*(000) = 712
*M**_r_* = 338.31	*D*_x_ = 1.392 Mg m^−^^3^
Monoclinic, *C*2	Mo *K*α radiation, λ = 0.71073 Å
*a* = 11.3928 (8) Å	Cell parameters from 5033 reflections
*b* = 6.6476 (4) Å	θ = 2.8–27.9°
*c* = 21.4219 (13) Å	µ = 0.11 mm^−^^1^
β = 95.801 (3)°	*T* = 296 K
*V* = 1614.07 (18) Å^3^	Block, colourless
*Z* = 4	0.30 × 0.20 × 0.20 mm

### Data collection

**Table:**

Bruker Kappa APEXII CCD diffractometer	2731 reflections with *I* > 2σ(*I*)
Radiation source: Sealed X-ray tube	*R*_int_ = 0.025
ω and φ scan	θ_max_ = 26.0°, θ_min_ = 2.9°
Absorption correction: multi-scan (*SADABS*; Bruker, 2004)	*h* = −14→14
*T*_min_ = 0.898, *T*_max_ = 0.978	*k* = −8→8
10737 measured reflections	*l* = −26→26
3157 independent reflections	

### Refinement

**Table:**

Refinement on *F*^2^	Secondary atom site location: difference Fourier map
Least-squares matrix: full	Hydrogen site location: mixed
*R*[*F*^2^ > 2σ(*F*^2^)] = 0.034	H atoms treated by a mixture of independent and constrained refinement
*wR*(*F*^2^) = 0.084	*w* = 1/[σ^2^(*F*_o_^2^) + (0.0458*P*)^2^ + 0.2154*P*] where *P* = (*F*_o_^2^ + 2*F*_c_^2^)/3
*S* = 1.04	(Δ/σ)_max_ < 0.001
3157 reflections	Δρ_max_ = 0.18 e Å^−^^3^
242 parameters	Δρ_min_ = −0.15 e Å^−^^3^
4 restraints	Extinction correction: *SHELXL2014* (Sheldrick, 2015), Fc^*^=kFc[1+0.001xFc^2^λ^3^/sin(2θ)]^-1/4^
Primary atom site location: structure-invariant direct methods	Extinction coefficient: 0.0069 (11)

### Special details

**Table:**

Geometry. All esds (except the esd in the dihedral angle between two l.s. planes) are estimated using the full covariance matrix. The cell esds are taken into account individually in the estimation of esds in distances, angles and torsion angles; correlations between esds in cell parameters are only used when they are defined by crystal symmetry. An approximate (isotropic) treatment of cell esds is used for estimating esds involving l.s. planes.

### Fractional atomic coordinates and isotropic or equivalent isotropic displacement parameters (Å^2^)

**Table:**

	*x*	*y*	*z*	*U*_iso_*/*U*_eq_	
N1	0.75082 (19)	0.1813 (4)	0.77433 (10)	0.0380 (5)	
H1	0.825 (3)	0.191 (5)	0.7735 (13)	0.047 (8)*	
N2	0.5095 (2)	0.4264 (3)	0.91895 (10)	0.0276 (5)	
H2A	0.472 (3)	0.534 (5)	0.9350 (15)	0.056 (10)*	
H2B	0.563 (3)	0.476 (5)	0.8921 (14)	0.049 (8)*	
H2C	0.554 (3)	0.365 (4)	0.9546 (15)	0.047 (9)*	
O1	0.24153 (16)	0.1425 (3)	0.89730 (8)	0.0483 (5)	
O2	0.35274 (17)	0.2275 (3)	0.98224 (8)	0.0485 (6)	
O3	0.50562 (13)	0.6533 (3)	0.80307 (7)	0.0334 (4)	
O4	0.69014 (13)	0.6700 (3)	0.77851 (7)	0.0381 (4)	
H4O	0.7050	0.6615	0.8167	0.057*	
O5	0.63962 (18)	0.6937 (5)	0.54724 (8)	0.0706 (7)	
H5O	0.6080	0.6855	0.5112	0.106*	
O6	0.45352 (18)	0.6884 (4)	0.57018 (8)	0.0595 (6)	
O7	0.37013 (19)	0.7265 (3)	0.97085 (11)	0.0532 (6)	
H7A	0.304 (2)	0.705 (5)	0.9880 (16)	0.080*	
H7B	0.382 (3)	0.852 (4)	0.9713 (18)	0.080*	
C1	0.3327 (2)	0.2159 (3)	0.92463 (10)	0.0263 (5)	
C2	0.4263 (2)	0.2860 (3)	0.88365 (10)	0.0244 (5)	
H2	0.3876	0.3556	0.8468	0.029*	
C3	0.4928 (2)	0.1034 (4)	0.86179 (12)	0.0328 (6)	
H3A	0.5345	0.0401	0.8983	0.039*	
H3B	0.4354	0.0073	0.8432	0.039*	
C4	0.5792 (2)	0.1458 (4)	0.81563 (11)	0.0302 (5)	
C5	0.6979 (2)	0.1602 (4)	0.82805 (11)	0.0357 (6)	
H5	0.7379	0.1562	0.8681	0.043*	
C6	0.6657 (2)	0.1816 (4)	0.72436 (11)	0.0333 (6)	
C7	0.6742 (2)	0.1930 (5)	0.66020 (12)	0.0445 (7)	
H7	0.7471	0.2041	0.6445	0.053*	
C8	0.5720 (3)	0.1874 (5)	0.62093 (12)	0.0508 (7)	
H8	0.5756	0.1951	0.5778	0.061*	
C9	0.4630 (2)	0.1705 (5)	0.64405 (13)	0.0507 (7)	
H9	0.3951	0.1677	0.6160	0.061*	
C10	0.4529 (2)	0.1579 (5)	0.70688 (11)	0.0417 (6)	
H10	0.3792	0.1472	0.7216	0.050*	
C11	0.5557 (2)	0.1615 (4)	0.74880 (11)	0.0312 (5)	
C13	0.57628 (19)	0.6648 (4)	0.76415 (10)	0.0286 (5)	
C14	0.5377 (2)	0.6733 (4)	0.69680 (10)	0.0365 (6)	
H14	0.4570	0.6672	0.6848	0.044*	
C15	0.6070 (2)	0.6888 (5)	0.65258 (11)	0.0418 (7)	
H15	0.6880	0.6994	0.6632	0.050*	
C16	0.5603 (2)	0.6899 (5)	0.58582 (11)	0.0447 (7)	

### Atomic displacement parameters (Å^2^)

**Table:**

	*U*^11^	*U*^22^	*U*^33^	*U*^12^	*U*^13^	*U*^23^
N1	0.0245 (11)	0.0520 (14)	0.0381 (12)	0.0037 (12)	0.0067 (9)	−0.0028 (12)
N2	0.0274 (12)	0.0345 (12)	0.0219 (11)	−0.0020 (9)	0.0072 (9)	0.0000 (9)
O1	0.0374 (11)	0.0818 (15)	0.0254 (9)	−0.0268 (11)	0.0019 (7)	−0.0006 (10)
O2	0.0441 (11)	0.0834 (16)	0.0183 (9)	−0.0273 (11)	0.0045 (7)	−0.0019 (9)
O3	0.0300 (9)	0.0449 (10)	0.0260 (8)	−0.0024 (9)	0.0056 (7)	0.0031 (8)
O4	0.0278 (9)	0.0651 (11)	0.0216 (8)	−0.0034 (10)	0.0026 (6)	0.0003 (10)
O5	0.0548 (13)	0.133 (2)	0.0237 (9)	−0.0004 (17)	0.0058 (9)	−0.0012 (15)
O6	0.0464 (12)	0.1044 (17)	0.0265 (9)	0.0027 (13)	−0.0027 (8)	0.0012 (12)
O7	0.0511 (13)	0.0543 (13)	0.0578 (13)	0.0096 (11)	0.0227 (10)	0.0010 (11)
C1	0.0249 (12)	0.0323 (13)	0.0220 (11)	−0.0021 (10)	0.0034 (10)	−0.0003 (10)
C2	0.0250 (13)	0.0331 (12)	0.0149 (11)	0.0015 (10)	0.0011 (9)	0.0015 (9)
C3	0.0399 (15)	0.0318 (13)	0.0275 (13)	0.0034 (11)	0.0073 (12)	−0.0007 (10)
C4	0.0304 (12)	0.0315 (12)	0.0295 (12)	0.0041 (12)	0.0074 (9)	−0.0047 (11)
C5	0.0371 (14)	0.0387 (13)	0.0312 (13)	0.0068 (13)	0.0029 (10)	−0.0028 (12)
C6	0.0327 (13)	0.0365 (13)	0.0317 (12)	0.0040 (12)	0.0076 (10)	−0.0033 (12)
C7	0.0416 (15)	0.0558 (17)	0.0384 (14)	0.0055 (15)	0.0158 (12)	−0.0013 (14)
C8	0.0600 (19)	0.0640 (19)	0.0291 (13)	0.0095 (18)	0.0084 (13)	0.0003 (15)
C9	0.0446 (16)	0.0678 (19)	0.0374 (14)	0.0066 (17)	−0.0071 (12)	−0.0061 (16)
C10	0.0301 (13)	0.0578 (16)	0.0373 (14)	0.0054 (15)	0.0033 (11)	−0.0047 (15)
C11	0.0286 (12)	0.0331 (12)	0.0323 (12)	0.0049 (12)	0.0056 (9)	−0.0043 (12)
C13	0.0286 (12)	0.0313 (12)	0.0257 (11)	−0.0016 (12)	0.0024 (9)	0.0003 (11)
C14	0.0328 (13)	0.0500 (15)	0.0263 (12)	−0.0002 (14)	0.0002 (10)	−0.0013 (14)
C15	0.0339 (14)	0.0630 (18)	0.0278 (13)	0.0024 (15)	−0.0002 (11)	−0.0004 (14)
C16	0.0429 (16)	0.0637 (19)	0.0279 (13)	0.0031 (16)	0.0053 (11)	−0.0018 (15)

### Geometric parameters (Å, º)

**Table:**

N1—C5	1.359 (3)	C3—H3A	0.9700
N1—C6	1.370 (3)	C3—H3B	0.9700
N1—H1	0.85 (3)	C4—C5	1.355 (3)
N2—C2	1.482 (3)	C4—C11	1.433 (3)
N2—H2A	0.92 (4)	C5—H5	0.9300
N2—H2B	0.94 (3)	C6—C7	1.390 (3)
N2—H2C	0.96 (3)	C6—C11	1.413 (3)
O1—C1	1.240 (3)	C7—C8	1.366 (4)
O2—C1	1.235 (3)	C7—H7	0.9300
O3—C13	1.219 (3)	C8—C9	1.387 (4)
O4—C13	1.303 (3)	C8—H8	0.9300
O4—H4O	0.8200	C9—C10	1.365 (4)
O5—C16	1.285 (3)	C9—H9	0.9300
O5—H5O	0.8200	C10—C11	1.401 (3)
O6—C16	1.229 (3)	C10—H10	0.9300
O7—H7A	0.88 (2)	C13—C14	1.466 (3)
O7—H7B	0.85 (2)	C14—C15	1.297 (3)
C1—C2	1.521 (3)	C14—H14	0.9300
C2—C3	1.529 (3)	C15—C16	1.475 (3)
C2—H2	0.9800	C15—H15	0.9300
C3—C4	1.491 (3)		
			
C5—N1—C6	108.8 (2)	N1—C5—H5	124.4
C5—N1—H1	123.6 (19)	N1—C6—C7	131.2 (2)
C6—N1—H1	127.6 (19)	N1—C6—C11	107.1 (2)
C2—N2—H2A	113 (2)	C7—C6—C11	121.7 (2)
C2—N2—H2B	109.4 (19)	C8—C7—C6	117.9 (2)
H2A—N2—H2B	108 (3)	C8—C7—H7	121.1
C2—N2—H2C	113.4 (17)	C6—C7—H7	121.1
H2A—N2—H2C	105 (2)	C7—C8—C9	121.4 (2)
H2B—N2—H2C	108 (2)	C7—C8—H8	119.3
C13—O4—H4O	109.5	C9—C8—H8	119.3
C16—O5—H5O	109.5	C10—C9—C8	121.6 (3)
H7A—O7—H7B	107 (3)	C10—C9—H9	119.2
O2—C1—O1	123.9 (2)	C8—C9—H9	119.2
O2—C1—C2	119.2 (2)	C9—C10—C11	118.9 (2)
O1—C1—C2	116.79 (19)	C9—C10—H10	120.6
N2—C2—C1	110.35 (18)	C11—C10—H10	120.6
N2—C2—C3	110.2 (2)	C10—C11—C6	118.6 (2)
C1—C2—C3	109.37 (19)	C10—C11—C4	134.3 (2)
N2—C2—H2	109.0	C6—C11—C4	107.1 (2)
C1—C2—H2	109.0	O3—C13—O4	123.4 (2)
C3—C2—H2	109.0	O3—C13—C14	121.5 (2)
C4—C3—C2	115.7 (2)	O4—C13—C14	115.07 (19)
C4—C3—H3A	108.4	C15—C14—C13	125.3 (2)
C2—C3—H3A	108.4	C15—C14—H14	117.4
C4—C3—H3B	108.4	C13—C14—H14	117.4
C2—C3—H3B	108.4	C14—C15—C16	121.5 (2)
H3A—C3—H3B	107.4	C14—C15—H15	119.3
C5—C4—C11	105.8 (2)	C16—C15—H15	119.3
C5—C4—C3	126.6 (2)	O6—C16—O5	124.5 (2)
C11—C4—C3	127.4 (2)	O6—C16—C15	121.0 (2)
C4—C5—N1	111.1 (2)	O5—C16—C15	114.6 (2)
C4—C5—H5	124.4		
			
O2—C1—C2—N2	−21.1 (3)	C8—C9—C10—C11	−0.3 (5)
O1—C1—C2—N2	161.6 (2)	C9—C10—C11—C6	1.1 (4)
O2—C1—C2—C3	100.3 (3)	C9—C10—C11—C4	−177.8 (3)
O1—C1—C2—C3	−77.0 (3)	N1—C6—C11—C10	179.9 (3)
N2—C2—C3—C4	−64.7 (3)	C7—C6—C11—C10	−1.5 (4)
C1—C2—C3—C4	173.8 (2)	N1—C6—C11—C4	−0.9 (3)
C2—C3—C4—C5	101.2 (3)	C7—C6—C11—C4	177.6 (3)
C2—C3—C4—C11	−84.9 (3)	C5—C4—C11—C10	180.0 (3)
C11—C4—C5—N1	−0.7 (3)	C3—C4—C11—C10	5.1 (5)
C3—C4—C5—N1	174.2 (2)	C5—C4—C11—C6	1.0 (3)
C6—N1—C5—C4	0.1 (3)	C3—C4—C11—C6	−173.9 (2)
C5—N1—C6—C7	−177.9 (3)	O3—C13—C14—C15	178.9 (3)
C5—N1—C6—C11	0.5 (3)	O4—C13—C14—C15	−1.3 (4)
N1—C6—C7—C8	179.2 (3)	C13—C14—C15—C16	178.1 (3)
C11—C6—C7—C8	1.0 (4)	C14—C15—C16—O6	4.4 (5)
C6—C7—C8—C9	−0.1 (5)	C14—C15—C16—O5	−175.9 (3)
C7—C8—C9—C10	−0.3 (5)		

### Hydrogen-bond geometry (Å, º)

**Table:**

*D*—H···*A*	*D*—H	H···*A*	*D*···*A*	*D*—H···*A*
N1—H1···O3^i^	0.85 (3)	2.11 (3)	2.912 (3)	158 (3)
N2—H2*A*···O7	0.92 (4)	1.94 (4)	2.845 (3)	170 (3)
N2—H2*B*···O1^ii^	0.94 (3)	2.30 (3)	3.085 (3)	140 (2)
N2—H2*B*···O3	0.94 (3)	2.28 (3)	2.901 (3)	123 (2)
N2—H2*C*···O2^iii^	0.96 (3)	1.87 (3)	2.832 (3)	174 (3)
O4—H4*O*···O1^ii^	0.82	1.74	2.559 (2)	178
O5—H5*O*···O6^iv^	0.82	1.81	2.630 (3)	174
O7—H7*A*···O1^v^	0.88 (2)	2.60 (3)	3.261 (3)	133 (3)
O7—H7*A*···O2^v^	0.88 (2)	1.97 (2)	2.824 (3)	165 (3)
O7—H7*B*···O2^vi^	0.85 (2)	2.53 (3)	3.347 (3)	162 (3)
C3—H3*B*···O3^vii^	0.97	2.66	3.255 (3)	120
C5—H5···O7^i^	0.93	2.58	3.491 (3)	166

Symmetry codes: (i) *x*+1/2, *y*−1/2, *z*; (ii) *x*+1/2, *y*+1/2, *z*; (iii) −*x*+1, *y*, −*z*+2; (iv) −*x*+1, *y*, −*z*+1; (v) −*x*+1/2, *y*+1/2, −*z*+2; (vi) *x*, *y*+1, *z*; (vii) *x*, *y*−1, *z*.
