# Supplementary figures and images for: Crystal structure of l-tryptophan–fumaric acid–water (1/1/1)
Source: Acta Crystallogr E Crystallogr Commun. 2015 Aug 15;71(Pt 9):o661–2. doi: 10.1107/S205698901501484X (PMC4555439; doi:10.1107/S205698901501484X)

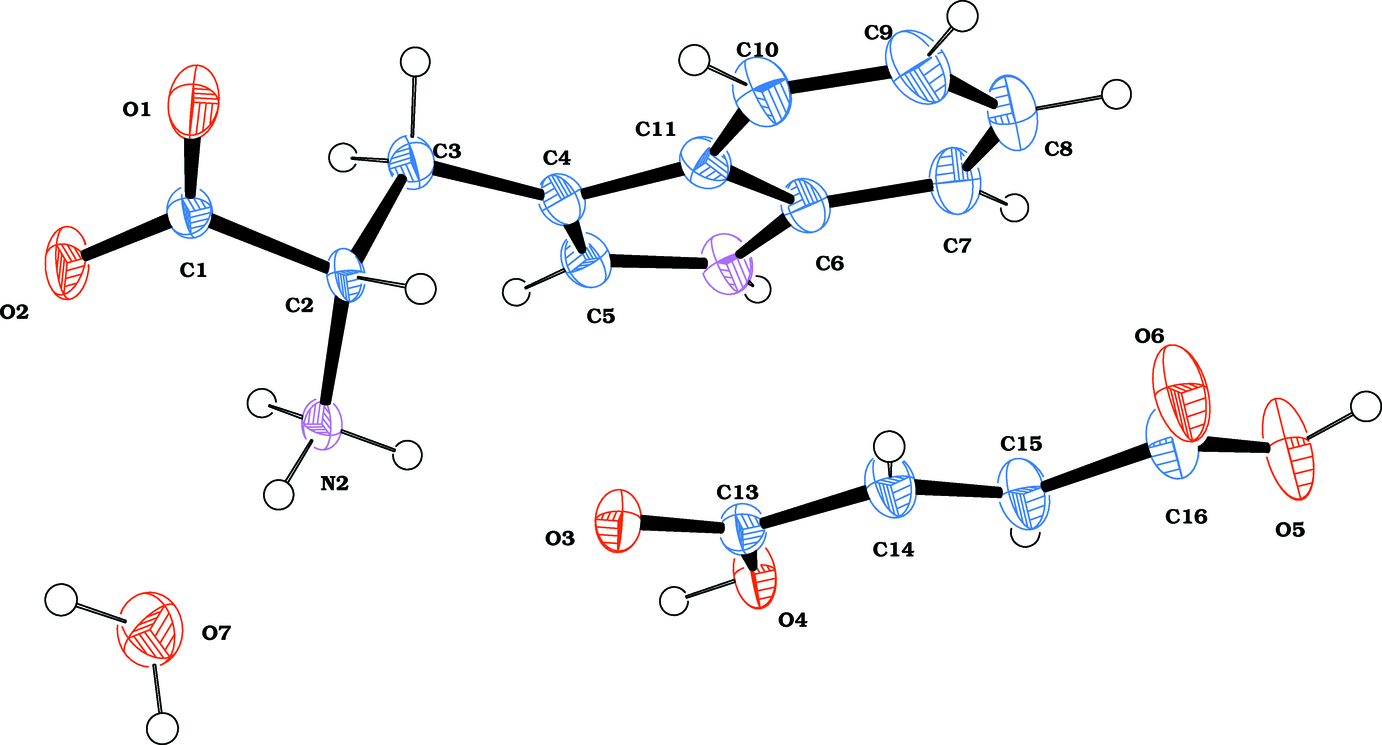

Supplement: Supplementary file 4 [file e-71-0o661-fig1.tif]

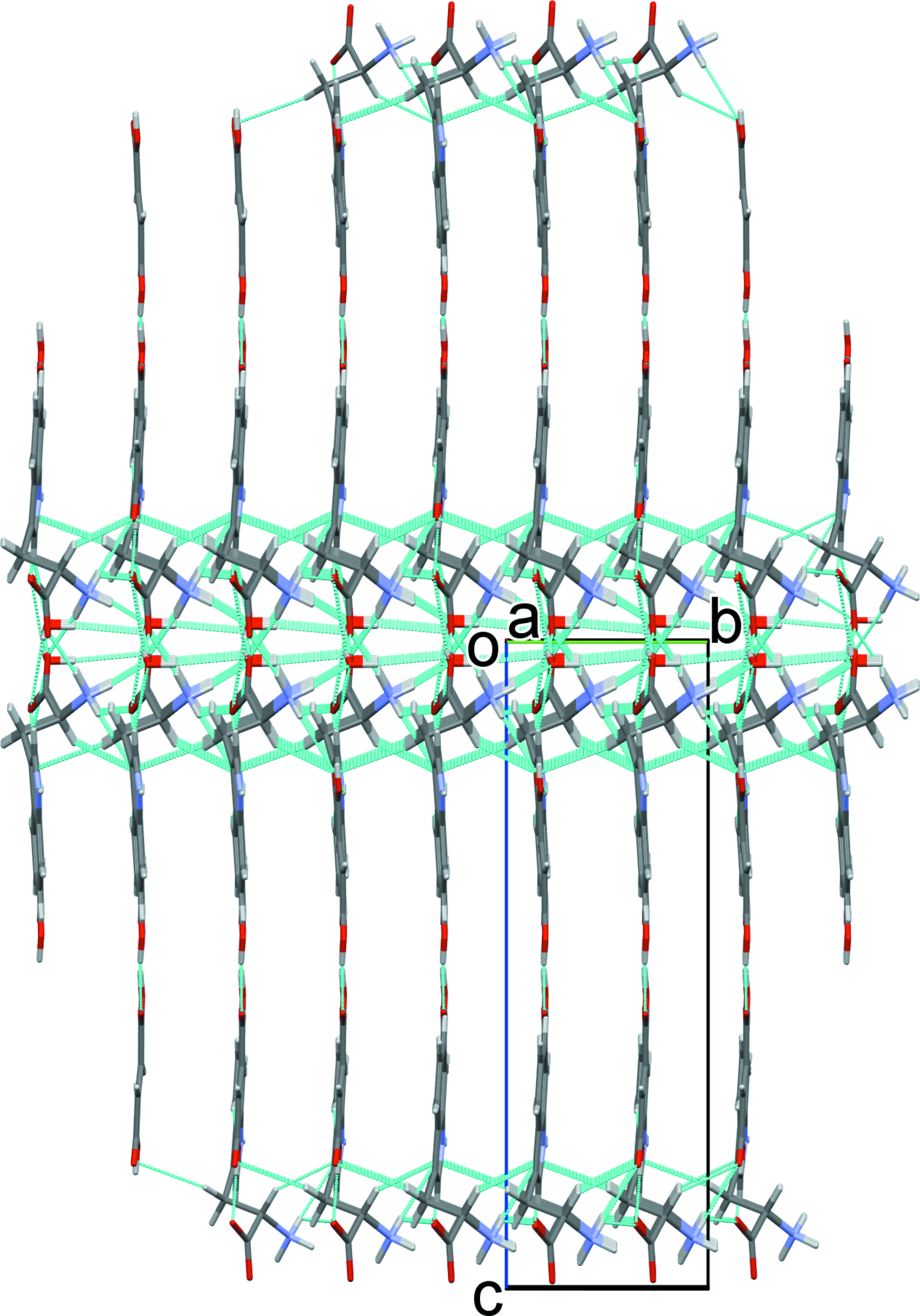

Supplement: Supplementary file 5 [file e-71-0o661-fig2.tif]

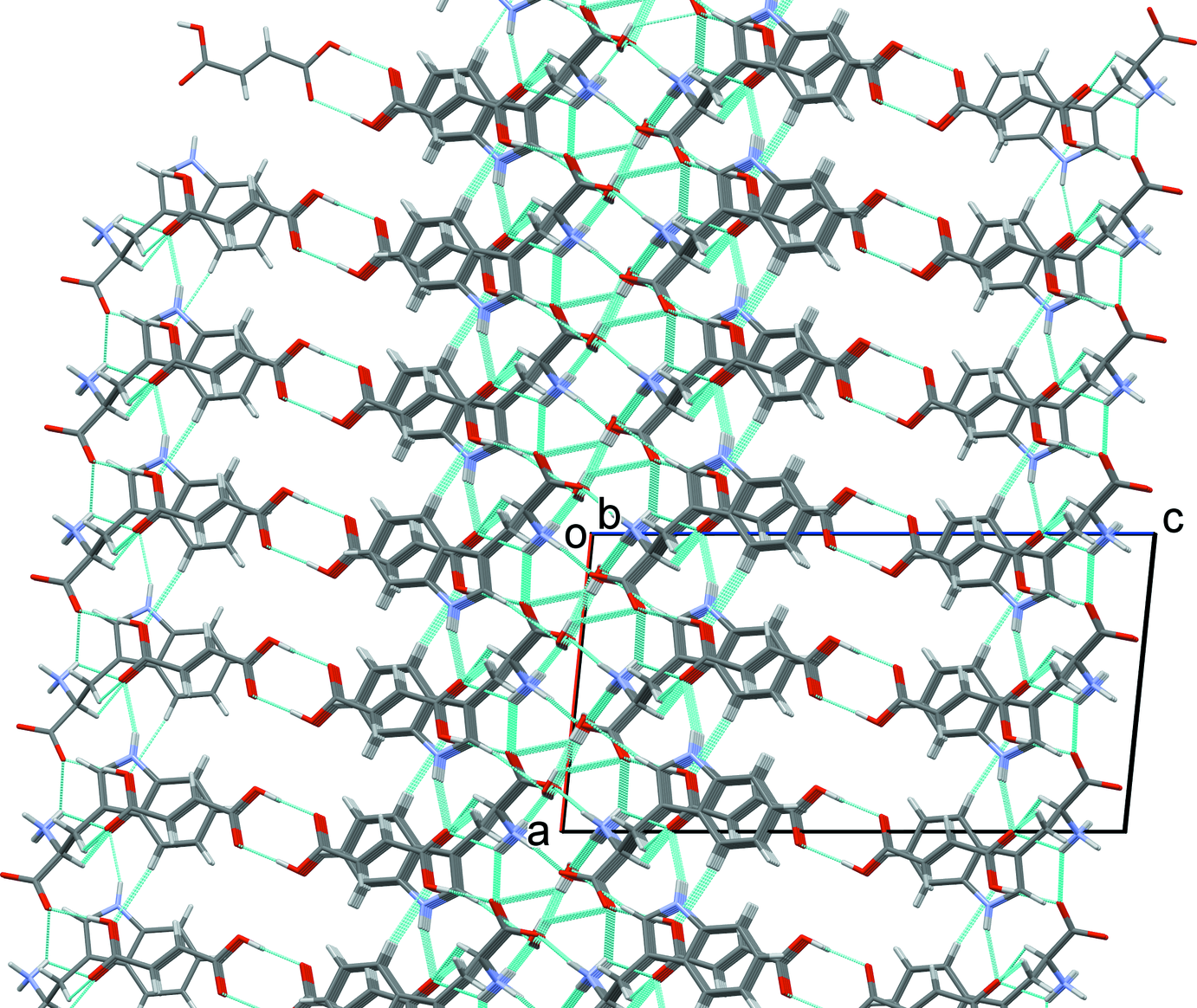

Supplement: Supplementary file 6 [file e-71-0o661-fig3.tif]
